# Prevention of Chronic Morbidities in Extremely Premature Newborns with LISA-nCPAP Respiratory Therapy and Adjuvant Perinatal Strategies

**DOI:** 10.3390/antiox12061149

**Published:** 2023-05-24

**Authors:** Gergely Balázs, András Balajthy, István Seri, Thomas Hegyi, Tibor Ertl, Tamás Szabó, Tamás Röszer, Ágnes Papp, József Balla, Tamás Gáll, György Balla

**Affiliations:** 1 Department of Pediatrics, Faculty of Medicine, University of Debrecen, 4032 Debrecen, Hungary; 2 First Department of Pediatrics, School of Medicine, Semmelweis University, 1083 Budapest, Hungary; 3 Keck School of Medicine of USC, Children’s Hospital of Los Angeles, Los Angeles, CA 90033, USA; 4 Department of Pediatrics, Division of Neonatology, Robert Wood Johnson Medical School, Rutgers, The State University of New Jersey, New Brunswick, NJ 08903, USA; 5 Departments of Neonatology and Obstetrics & Gynecology, University of Pécs Medical School, 7624 Pécs, Hungary; 6 MTA-PTE Human Reproduction Scientific Research Group, University of Pécs, 7624 Pécs, Hungary; 7 Department of Internal Medicine, Division of Nephrology, Faculty of Medicine, University of Debrecen, 4032 Debrecen, Hungary; 8 ELKH-UD Vascular Pathophysiology Research Group, Hungarian Academy of Sciences, University of Debrecen, 4032 Debrecen, Hungary

**Keywords:** LISA, NIV, prematurity, BPD, ROP, kidney, angiogenesis, NAC, insulin, antioxidants

## Abstract

Less invasive surfactant administration techniques, together with nasal continuous airway pressure (LISA-nCPAP) ventilation, an emerging noninvasive ventilation (NIV) technique in neonatology, are gaining more significance, even in extremely premature newborns (ELBW), under 27 weeks of gestational age. In this review, studies on LISA-nCPAP are compiled with an emphasis on short- and long-term morbidities associated with prematurity. Several perinatal preventative and therapeutic investigations are also discussed in order to start integrated therapies as numerous organ-saving techniques in addition to lung-protective ventilations. Two thirds of immature newborns can start their lives on NIV, and one third of them never need mechanical ventilation. With adjuvant intervention, these ratios are expected to be increased, resulting in better outcomes. Optimized cardiopulmonary transition, especially physiologic cord clamping, could have an additively beneficial effect on patient outcomes gained from NIV. Organ development and angiogenesis are strictly linked not only in the immature lung and retina, but also possibly in the kidney, and optimized interventions using angiogenic growth factors could lead to better morbidity-free survival. Corticosteroids, caffeine, insulin, thyroid hormones, antioxidants, N-acetylcysteine, and, moreover, the immunomodulatory components of mother’s milk are also discussed as adjuvant treatments, since immature newborns deserve more complex neonatal interventions.

## 1. Introduction

Infants born before 28 weeks of pregnancy are considered extremely preterm [1]. According to the World Health Organization (WHO), an extremely-low-birth-weight (ELBW) infant is defined as one with a birth weight of less than 1000 g. Most extremely-low-birth-weight infants are also the youngest of premature newborns, usually born at 27 weeks gestational age or less.

They represent a minority of preterm births and account for only ~0.5% of all births [1], also contributing disproportionally to NICU deaths [2]. Although mortality continues to decrease in this population, the incidence of long-term complications, including bronchopulmonary dysplasia (BPD), intraventricular hemorrhage (IVH), and retinopathy of prematurity (ROP), remains unacceptably high [1,3,4,5]. For this reason, in addition to the stabilization of basic life functions, it is particularly important that in the care of the most vulnerable patients, sufficient attention should also be paid to the prevention of these diseases. The introduction of less invasive surfactant administration (LISA) combined with nasal continuous positive airway pressure (nCPAP) support as a part of a complex care bundle may improve chronic morbidity-free survival in extremely preterm infants. LISA combined with nasal continuous positive airway pressure (LISA-nCPAP) respiratory treatment, a rediscovered form of NIV techniques, exerts the most significant protective effects on the prevention of lung volutrauma/barotrauma [6], and reduces the need for mechanical ventilation [7,8,9]. Accumulating evidence proves that LISA reduces the death rate and BPD frequency compared to surfactant delivery via intubation [9,10]. 

The intrauterine milieu of the fetus is characterized by a sensitive balance of inflammatory and anti-inflammatory processes, vascular growth factors and oxygen-stress-free environment which fundamentally changes after preterm delivery.

The aim of this narrative review is to summarize the current evidence on LISA and adjuvant perinatal strategies, including these relevant sections:

- cardiopulmonary transition
- angiogenesis
- antioxidants
- nephrogenesis and renal function
- glucose metabolism
- inflammation.

## 2. Less Invasive Surfactant Administration (LISA) Combined with Nasal Continuous Positive Airway Pressure (nCPAP) Respiratory Therapy in Clinical Practice

Due to the findings of large randomized controlled trials (RCT) investigating the treatment of respiratory distress syndrome (RDS), the prophylactic administration of surfactant via endotracheal intubation has been replaced by the use of early CPAP, thereby avoiding mechanical ventilation [11,12,13]. However, CPAP alone fails to provide sufficient support in 40–65% of preterm infants within the first 72 h of life; these patients require delayed endotracheal intubation followed by mechanical ventilation, which is associated with a significant risk of complications and long-term morbidities [14,15,16]. Considering the consequences of delayed therapy, many neonatologists often decide to perform early endotracheal intubation to ensure the benefits of exogenous surfactant therapy [17]. Which one is more beneficial: to treat premature infants with non-invasive respiratory support with the risk of CPAP failure (defined as the need for intubation before 72 h of life), or to accept the complications of intubation and invasive ventilation required for early surfactant therapy? Is it possible to implement the two procedures together? LISA-nCPAP respiratory treatment provides a solution.

In 1992, a thin catheter method for surfactant administration was described for the first time [18], and the technique was rediscovered a decade later by Kribs et al. [19]. During LISA, a thin catheter is introduced into the trachea under direct laryngoscopy for surfactant delivery, while nCPAP is continued without interruption, and the infant can breathe spontaneously [20]. The main advantage of the procedure compared to the administration of the surfactant through an endotracheal tube is the avoidance of invasive ventilation. While LISA used to be a stand-alone intervention, today it forms part of a complex LISA-nCPAP care bundle supporting the transition to extra-uterine life, which includes antenatal steroid prophylaxis, delivery room care interventions such as delayed or physiologic cord clamping, temperature control, minimal handling approach, tactile stimulation, early prophylactic use of nCPAP at higher (≥9 cmH_2_O) pressures, early caffeine loading, and skin-to-skin contact [21,22]. 

Since its first description, various flexible and semi-rigid catheter techniques have been reported for LISA [20,23], and the preferred choice of equipment shows significant differences worldwide [24,25,26,27,28,29]. According to clinical trials [30,31] and in contrast with simulation studies [32,33], experienced neonatologists are able to perform endotracheal catheterization using both semi-rigid and flexible catheters at similar rates and ease.

Table 1 presents the results of LISA-related RCTs [34,35,36,37,38,39,40,41,42,43,44,45,46,47,48,49,50,51,52], while Table 2 summarises meta-analyses comparing LISA with surfactant administration via an endotracheal tube (S-ETT), with or without extubation (INSURE or infants remained intubated after surfactant delivery) [10,53,54,55,56,57]. Although there are still many unanswered questions regarding the use of LISA-nCPAP support, the latest European Consensus Guidelines on the Management of respiratory distress syndrome recommends LISA as the preferred mode of surfactant administration for spontaneously breathing infants on CPAP [58].

Although the introduction of LISA has increased the success rate of early prophylactic CPAP, 23–62% of preterm infants undergoing LISA still require intubation and mechanical ventilation during their first 72 h of life (LISA failure) [35,59,60,61,62]. In their retrospective study, Janssen et al. identified four independent factors associated with LISA failure: gestational age less than 28 weeks at birth, elevated C-reactive protein (CRP) levels 24 h after birth, lack of antenatal steroid (ANS) prophylaxis, and surfactant dose less than 200 mg/kg [60]. With a post hoc analysis of their prospective cohort study, Kruczek et al. found that FiO_2_ was an independent risk factor for LISA failure [63], while Ramos-Navarro et al. noticed that a reduction in FiO_2_ after LISA was a predictor of treatment success (defined as no need for intubation during the first 72 h of life) [62]. In their retrospective observational study, investigating the short-term outcome after repeated LISA (reLISA), Kleijkers et al. found that reLISA is effective in reducing CPAP failure, and is associated with a lower risk of death or BPD compared to retreatment via an endotracheal tube [64]. One of the most important questions regarding the use of LISA is how to identify preterm infants whose RDS can be successfully treated with a single LISA. Therefore, unlike other reports already available surrounding the topic [60,63], we have put emphasis on predicting the success of the first LISA treatment, and subsequently, we have defined LISA failure as the need of a second intervention (another LISA) and/or mechanical ventilation within the first 72 h of life. We found that LISA success (defined as no need for additional surfactant treatment and/or mechanical ventilation within 72 h after the first LISA) can be predicted by variables available before the intervention. The independent predictors of the outcome of the first LISA are the birth weight, maternal age, core temperature at the time of admission, highest respiratory severity score during the first hour of life or at the time of LISA, dose of poractant alfa, and the level of CRP [65].

The amount of data available regarding the effect of LISA-nCPAP on long-term outcomes is limited. To date, the results of two RCT follow-up studies have been published. They suggest that LISA-nCPAP support can result in an unchanged or even better neurological outcome compared to surfactant administration via ETT with delayed intubation [8,66].

### 2.1. Timing and Thresholds of Treatment

Considering the pathophysiology of RDS, exogenous surfactant replacement should be performed as soon as possible. Compared to rescue administration, early prophylactic surfactant therapy (≤2 h of life) resulted in better survival in mechanically ventilated preterm infants [67]. Among the RCTs investigating LISA, very early (<120 min of life) treatment was performed in the NINSAPP trial examining infants born before 27 weeks gestation, while the average time of surfactant treatment in the other studies was between 45 min and 15.4 h, presumably determined by the applied FiO_2_ thresholds [34,36,37,38,39,41,43,45,46,47,48,49,50]. Data from the German Neonatal Network shows a tendency for LISA to be performed earlier and earlier (at 20–30 min of life), but no RCTs are available yet regarding this “quasi-prophylactic” administration of surfactant [22,61].

Several observational studies have confirmed that FiO_2_ levels >0.3 predict early CPAP failure associated with adverse events, and it is therefore recommended that the treatment threshold of FiO_2_ >0.30 is used for all preterm infants diagnosed with RDS [58]. However, although FiO_2_ serves as a good screening tool, it does not characterize the patients’ oxygenation in sufficient detail. For this reason, it is a challenge to identify those preterm infants who would benefit most from early, selective surfactant therapy, within 3 h of life. Furthermore, it is extremely important to identify the patients that will not benefit from a non-invasive approach. It would therefore be appropriate to develop a personalized, physiology-driven treatment for RDS instead of the existing uniform treatment strategy [68]. Various diagnostic methods that estimate the endogenous surfactant pool could help to solve this problem [69,70,71,72,73,74,75], but to date, these methods are limited to research use and have not been used in everyday practice. The saturation oxygen pressure index (SOPI), calculated as CPAP pressure x FiO_2_/SpO_2_, can be an excellent non-invasive monitoring tool for the diagnosis and progression of RDS; however, further studies are required for its widespread application. Preterm infants failing on CPAP during the first 72 h of life require a higher FiO_2_ and CPAP pressure, and more positive pressure ventilation cycles during delivery room stabilization [76]. In a prospective, observational, non-randomized study, lung reactance (Xrs) within 2 h after birth assessed by the forced oscillation technique (FOT) could identify the infants who would need surfactant within 24 h [77]. 

It is well known that lung ultrasound (LUS) is a reliable tool for the early diagnosis of RDS. This modality is one of the most promising methods for individualizing the treatment of RDS [68,78,79]. In preterm infants, the lung ultrasound score is calculated based on ultrasound abnormalities in three different regions per lung, and correlates well with oxygenation status, predicting the severity of RDS and subsequent need for surfactant treatment, as well as CPAP failure [80,81,82,83]. De Luca et al. found that LUS improved the timing of surfactant administration [84,85]. A single RCT, involving preterm infants born before 32 weeks of gestation, demonstrated that the ultrasound-guided LISA group received surfactant earlier (1 vs. 6 h of life, *p* < 0.001) and at a lower FiO_2_ exposure compared to the control patients (25% vs. 30%, *p* = 0.012) [86].

### 2.2. Premedication for LISA-nCPAP

A significant heterogeneity can be seen worldwide in premedication [24,26,27,28,87]. The use of atropine was associated with a significantly lower incidence of bradycardia [88]. Adequate sedation ensures not only patient comfort, but it can also reduce the incidence of adverse events and might increase the success rate of the procedure [89,90,91]. However, these drugs can have significant side effects, including prolonged apnoea requiring endotracheal intubation [92,93]. Non-pharmacological pain relief administered before LISA (positioning, swaddling, administration of buccal sucrose or breast milk) may significantly reduce the need for sedatives during the procedure [94]. Multiple studies have been conducted investigating the effect of various sedatives (propofol, fentanyl, ketamine, midazolam) with controversial results [87,91,92,93,95,96,97]. The use of sedatives does not affect the length of the intervention, the success rate of tracheal catheterization during the first laryngoscopic attempt, and the need for early intubation, but sedation may increase the need for nasal intermittent positive pressure, the risk of desaturation and apnea demonstrated by a meta-analysis based on 33 studies [90]. For most outcomes, the certainty of evidence is low/very low; therefore, further trials are warranted to explore the use of premedication for LISA. 

### 2.3. LISA-nCPAP Support in Extremely Preterm Infants Born before 27 Weeks of Gestation

There is no consensus regarding the use of LISA-nCPAP support among the most vulnerable group of extremely preterm infants [21,58], although this population needs lung-protective ventilation strategies the most. The NINSAPP trial (Table 1) involving preterm infants born between 23 and 26 weeks demonstrated that LISA is feasible in this population and its use was significantly more favorable compared to S-ETT [35,66].

In a large German Neonatal Network (GNN) cohort study of infants born between 22 and 29 weeks gestational age (*n* = 7533), LISA-nCPAP was associated with significantly lower rates of BPD, grade II–IV stages of IVH and ROP compared to surfactant treatment via ETT [98]. However, in the group under 26 weeks of gestation, the risk of focal intestinal perforation (FIP) was found to be higher. In a multivariable logistic regression analysis, compared to S-ETT, LISA was associated with an increased risk of FIP. However, other studies did not confirm this association [22,35,37].

During the OPTIMIST-A trial [37] (Table 1), death or BPD assessed at 36 weeks postmenstrual age occurred in 43.6% of infants in the LISA group and 49.6% in the control group (RR = 0.87, 95% CI 0.74–1.03, *p* = 0.1). There was no significant difference in mortality between the two groups, the incidence of BPD was significantly lower in the LISA group. A recent observational study of 6542 preterm infants born between 22 and 26 weeks conducted by GNN demonstrated that LISA exposure compared to infants without LISA exposure resulted in significantly lower all-cause death, BPD, and death or BPD rates [22]. More than 80% of extremely premature newborns with a gestational age between 22 and 26 weeks were treated with LISA in the delivery room, much earlier than in the OPTIMIST-A trial [22].

## 3. Optimized Perinatal Cardiopulmonary Transition Supports the Success of LISA-nCPAP Respiratory Therapy

By its noninvasive nature, LISA-nCPAP respiratory support is less likely to interfere with the process of early postnatal cardiopulmonary adaptation to the extent seen with invasive surfactant administration and, especially, invasive mechanical ventilation. A great number of data on LISA/NIV and their impact on neonatal hemodynamics, cardiovascular care, and outcomes originate from retrospective studies often using historical controls [61,99,100]. Nevertheless, many questions remain concerning LISA-nCPAP, especially since the outcomes associated with its use have primarily been compared to those of preterm neonates, mostly on prolonged invasive ventilation [101].

### 3.1. Physiology of Cardiopulmonary Transition

The physiology of the cardiopulmonary transition from fetal to postnatal life is an extremely complex process with tightly regulated and interdependent sequences of events. When discussing the physiology of cardiopulmonary transition in the context of NIV modalities, the first important issue that has to be addressed is the timing of umbilical cord clamping. It is also tempting to speculate that for LISA-CPAP to achieve its highest success rates, physiologic umbilical cord clamping needs to be practiced even in the extremely preterm neonate of 23–27 weeks gestation. To address this topic, the clinically most relevant physiological features of cardiopulmonary transition have to be discussed [102], followed by a summary of the hemodynamic responses to the use of NIV techniques. 

#### 3.1.1. Fetal Circulation

Throughout fetal life, the placenta serves as the organ of gas exchange in place of the lungs, and the systemic and pulmonary circulations function in a parallel manner [102]. The connections between the two circulations are provided via the right-to-left shunts through the foramen ovale (FO) and ductus arteriosus, enabling the provision of relatively well-oxygenated blood to the fetal organs and the delivery of deoxygenated blood to the placenta. In the fetus, the “oxygenated” blood from the umbilical vein with a hemoglobin O_2_ saturation of 83% and PO_2_ of 30–35 mmHg, enters the ductus venosus, mixes with the venous blood from the hepatic and portal veins, and enters the inferior vena cava (IVC). After mixing with the venous blood from the distal part of the IVC, blood enters the right atrium. Here, the majority of the IVC flow shoots across the FO into the left atrium. From there, after mixing with the deoxygenated, but limited volume of blood returning from the lungs via the pulmonary veins, blood enters the left ventricle, providing relatively higher oxygenated blood for the brain, with a hemoglobin O_2_ saturation of 60% and a PO_2_ of 20–25 mmHg. Thus, the vast majority of the left ventricular output is provided by the relatively well-oxygenated blood originating from the placenta. As for the right ventricular output, it is mostly supplied by the blood arriving back from the brain and the upper body via the superior vena cava (SVC) which enters the right atrium. In addition, a smaller portion also originates from the more oxygenated blood of the IVC not finding its way across the FO into the left atrium. However, due to the high pulmonary vascular resistance, the majority of the right ventricular output bypasses the lungs and flows from the main pulmonary artery through the wide-open ductus arteriosus into the descending aorta. Importantly, except for the mid-systole, blood flows away from the high-resistance pulmonary circulation, ensuring a continuous right-to-left ductal flow into the systemic circulation even throughout the diastole. 

Thus, in the fetal circulation, umbilical venous blood flow and SVC blood flow are the major sources of left and right ventricular preload, respectively [102]. This information is of critical importance for understanding the physiology of cardiopulmonary transition at birth. It also helps explain why the low cardiac output syndrome and the potentially ensuing peri/intraventricular hemorrhage (P/IVH) may develop in patients with an altered ventricular preload [103,104], especially when immediate cord clamping is practiced. The benefits of delayed cord clamping (defined as a 30 to 180 s delay in the clamping of the cord) include a somewhat decreased rate of all grades of P/IVH, decreased need for transfusion, improved cardiovascular stability, and a lower risk of death at discharge [105]. It must be emphasized, though, that the benefits of physiologic cord clamping have not yet been systematically investigated. 

As with physiologic cord clamping, the inappropriate use of invasive ventilation in very preterm neonates who might not need this type of support may alter cardiac loading conditions and thus cardiac function. This, in turn, will exert a negative effect on systemic and organ blood flow. Hence with the sequential utilization of physiologic cord clamping, LISA-nCPAP holds the promise of enhancing postnatal hemodynamic stability, and thus improving the short- and long-term outcomes such as P/IVH, periventricular leukomalacia (PVL), and the overall neurodevelopment. 

#### 3.1.2. Postnatal (Adult-Type) Circulation

At delivery, filling the lungs with air triggers the pulmonary and circulatory changes required for the onset of pulmonary gas exchange, along with the adaptive responses necessary for postnatal survival. These circulatory changes and the clamping of the umbilical cord at the appropriate time are ultimately responsible for the proper transitioning from the fetal, parallel circulatory pattern to the postnatal phenotype, where the pulmonary and systemic circulations are connected in series.

If the cord is clamped immediately at birth, it first results in an extremely rapid and brief increase in systemic vascular resistance and blood pressure, along with an unmitigated rise in cerebral blood flow (CBF). This is especially dangerous in the very preterm neonate with immature cerebral autoregulatory capacity and cerebral structural immaturity. In addition, as 30–50% of the total fetal cardiac output flows through the placenta, immediate cord clamping in the absence of lung aeration also results in an immense reduction in ventricular preload and output. Thus, the rapid and unmitigated rise in the CBF in patients undergoing immediate cord clamping and inappropriate lung aeration at delivery is followed by a significant drop in the CBF due to the decline in cardiac output and thus systemic blood flow. The systemic organ hypoperfusion then triggers an adaptive cardiovascular response, ensuring a certain level of protection of the vital organs, including the brain, heart, and adrenal glands [106]. Unfortunately, as the forebrain vasculature does not appear to have reached a vital organ assignment in the very preterm neonate [106], forebrain ischemia also develops. Later, as the cardiopulmonary condition improves, and organ reperfusion takes place, affecting the unprotected brain as well [104,107]. Indeed, this is the time when the majority of P/IVH occurs. One might speculate that interventions supporting rather than interfering with the process of postnatal transition, including the use of physiologic cord clamping, LISA-nCPAP, might exert some protective effect for these patients. 

On the other hand, if the lungs are aerated first and the umbilical cord is only clamped after the establishment of lung aeration, these changes attenuate the immediate increase followed by the decrease in CBF seen in neonates with immediate cord clamping and/or in the absence of appropriate lung inflation. Additionally, these changes ensure the establishment of appropriate pulmonary, systemic, and cerebral blood flow within minutes following delivery. Therefore, complications may be less likely to occur as, for instance, the identified cardiorespiratory antecedents of P/IVH are mitigated and expected to cause less harm in this extremely vulnerable patient population [104,107].

### 3.2. Clinically Relevant Cardiovascular Outcomes with the Use of LISA-nCPAP

A recent prospective study on a small number of preterm neonates with respiratory distress syndrome compared the effect of LISA-nCPAP to the surfactant given via the INSURE procedure on cerebral tissue oxygenation (CrSO_2_) and oxygen extraction assessed by near infrared spectroscopy (NIRS) [108]. Cerebral tissue oxygenation decreased with both procedures, and the decrease was larger with LISA-nCPAP even 2 h after the procedure. The clinical importance of the NIRS findings is unclear, especially in light of the findings of a recent retrospective study [100]. A latter study demonstrates a decreased need for FiO_2_ shortly after the completion of the LISA lasting for at least two hours [100]. Please note the retrospective nature, the use of historical controls, and the enrollment of more mature (27–32 weeks gestation) preterm neonates in this study [100]. Nevertheless, it is reasonable to expect that CrSO_2_ decreases to a certain degree during LISA-nCPAP, especially in very preterm neonates. Importantly, this study [100] has also found an association between the use of LISA-nCPAP and a decreased need for mechanical ventilation during the first three postnatal days (20.2% vs. 56.6%, *p* < 0.001), a drop in the incidence of moderate-to-severe BPD (8.2% vs. 20.2%, *p* = 0.02) and a decrease in the cost of neonatal intensive care and hospital stay. 

As for the hemodynamic effects of NIV, a study in 20 relatively stable preterm infants with a mean gestational age of 27 (25–32) weeks compares right and left ventricular output and anterior cerebral blood flow velocity between nCPAP and non-synchronized NIV at a median postnatal age of 20 (9–28) days [109]. Switching between the two modalities has no discernible impact on the hemodynamic parameters assessed. However, as the patient selection, type of device [110], sedation use, and clinical skills when performing LISA-nCPAP significantly influence the clinical effects of the procedure [89], more prospective studies on larger patient populations are clearly needed to investigate the impact of LISA-nCPAP on the cardiovascular system. 

## 4. Interaction of NIV/LISA-nCPAP Therapy with Angiogenesis and Organ Development

Improvement of the chronic morbidity-free survival rate as a result of the use of LISA-nCPAP respiratory support for extremely immature newborns largely depends on prenatal care as well. Universal prenatal steroid administration and antibiotic prevention help the cardiopulmonary transition, and the consequence is that two thirds of extremely premature newborns can avoid intubation and MV right after birth. 

### 4.1. Angiogenesis in the Perinatal Age

Hypoxia-inducible factors (HIFs) play an essential role in response to hypoxia. Hypoxia inhibits the degradation of HIF-1α, which controls the transcription of many proteins involved in the hypoxic response [111,112]. HIF-1α promotes the expression of several growth factors, including vascular endothelial growth factor (VEGF). VEGF plays a critical role in retinal neovascularization [113] and is linked to ROP development [114]. In addition to VEGF, several angiogenic factors, including angiopoietins (Angs), insulin-like growth factor-1 (IGF-1), and erythropoietin (Epo), are involved in the pathology of ROP.

In VEGF-induced postnatal neovascularization, evidence suggests that Ang-1 promotes vascular network maturation, whereas Ang-2 works to initiate neovessel formation [115]. The levels of vitreous Ang-2 in the eyes, with highly and moderately vascular-active ROP, are significantly higher than in control eyes [116]. 

The data on the relationship between ROP and Epo are contradictory. A retrospective cohort study shows that recombinant Epo exposure is independently associated with an increased risk of ROP progression [117], while others have found that early Epo administration does not increase the risk of ROP in preterm infants [118]. 

### 4.2. Agiogenesis and Vascular Growth Factors

Recombinant human insulin-like growth factor-1 (IGF-1) substitution significantly decreases the rate of BPD in premature newborns at the gestational age of 23–27 weeks [119]. In this phase 2 randomized, controlled trial, IGF-1 is delivered continuously until the gestational age of 29 weeks, and there is a tremendous 53% decrease in the rate of severe BPD incidence compared to the control group. More importantly, a subgroup analysis reveals that if the serum IGF-1 levels are within the target therapeutic range, there is an extreme 89% decrease in the rate of severe BPD, 4.8% vs. 44.9%. An exciting aspect of the study is that its primary aim is to prevent ROP. The disturbances of angiogenesis are the main issues in the pathophysiology of chronic morbidities in premature newborns, not only in BPD, but also in ROP, IVH, PVL, and moreover, in the delay of organ and entire body development. The fetal IGF-1 level increases during the second and third trimesters, which cannot happen in the case of immature births. This IGF-1 deficiency can be corrected by IGF-1 administration starting right after birth. The rate of ROP, greater than stage 3, increases in the IGF-1-treated group compared to the control, to 25.5% from 18%; the difference is not significant [119]. Importantly, there is a negative association between the serum IGF-1 concentration and ROP stages. The IVH results do not demonstrate significant differences between the IGF-1-treated and control groups, although there is a beneficial shift in the patterns of severity toward milder cases. Since this work does not present the side effects that would contraindicate further IGF-1 studies, one may suggest that the combination of NIV with IGF-1 could be promising for extremely premature newborns.

Right after birth, the metabolism turns into a more effective oxidative phosphorylation [120]. The initiator of this rapid metabolic change is the relatively high blood oxygen partial pressure, the intrauterine pressure is 30 mmHg compared to the extrauterine 95 mmHg. On the other hand, the higher oxygen concentration turns off many genes that are active throughout the intrauterine life, and provides normal organ development. The roles of VEGF and IGF-1 have been elegantly studied in the pathomechanism of ROP [121]. 

The oxygen is among the risk factors for ROP; the relationship is undeniable, since the milestones of ROP history are linked to neonatal oxygen therapy. Hyperoxia is a trigger for genetic and epigenetic alterations contributing to the development of ROP and BPD, and, perhaps to lifelong changes [122]. Although oxygen itself is a free radical, exogenous antioxidants do not have preventive or therapeutic effects on chronic neonatal morbidities. Contrary to that, angiogenic growth factors, closely related to oxygen metabolism, play significant roles in the pathomechanism of ROP. Intriguingly, one of the first IGF-1 lung studies shows that hyperoxia acts not only on VEGF, but also on the IGF-1 system by inhibiting IGF-1 from binding to its receptor, which hampers alveolar and vascular development [123].

The deficiency of vascular growth factors is the primary cause of the pathomechanism of lung hypoplasia [124,125]. Premature infants, who later develop BPD, have low serum VEGF levels in the first week of life [126]. Intravitreal anti-VEGF treatment is a part of the ROP therapeutic recommendation, safe and as effective as laser therapy [127]. In a retrospective study, a significantly longer respiratory therapy is associated with the anti-VEGF therapy [128]. Pulmonary hypertension is a good marker of pulmonary conditions, especially in extremely premature infants. Pulmonary hypertension can predict chronic morbidities, such as BPD [120]. An extremely small dose of anti-VEGF drug may act on lung development, since monoclonal anti-VEGF administration against ROP is associated with pulmonary hypertension [129].

### 4.3. Oxygen Therapy

The etiology of BPD in preterm neonates is strongly associated with oxygen therapy and ROS production, which injures the developing lungs [130,131]. Hyperoxia induces excessive production of reactive oxygen species, triggering oxidative stress and inflammation that contribute to pulmonary growth restriction and the inhibition of alveolarization and angiogenesis [132]. In addition, the induction of antioxidant defense is also impaired during hyperoxia [133,134]. Preterm infants exposed to intermittent hypoxic episodes as a consequence of immature respiratory control suffer from a proinflammatory cascade and ROS generation [133,135,136]. In an animal model, neonatal intermittent hypoxia/hyperoxia exposure induces long-term changes in the respiratory mechanics, and increases oxidative stress contributing to wheezing [137]. In double hit animal models, infection and hyperoxia aggravate the pro-inflammatory immune response and disrupt lung development [138,139,140]. Others have found that both hyperoxia and hypoxia, together with subsequent LPS stimulation, promote the pro-inflammatory response of preterm macrophages [141].

LISA-nCPAP respiratory therapy focuses not only on avoiding mechanical trauma of the lungs, but also on optimizing the dose and the length of oxygen therapy to minimize gene modifications, the consequence of oxygen metabolism, and free radical stress.

### 4.4. Oxidative Stress in Organ Development

The improving prenatal care, the increasingly widely used lung-protective respiratory therapy, and the strictly controlled oxygen supplementation drive neonatology into a new era when the old drugs among exogenous antioxidants may add some benefits to the prevention and treatment of chronic neonatal morbidities. 

Successfully applied LISA-nCPAP ventilatory therapy eliminates the tremendous volume and barotrauma of immature lungs, a robust detrimental iatrogenic complication of neonatal intensive care. Focusing on oxygen therapy, the same observation can be made in the field of oxygen toxicity. Oxygen toxicity consists of two basic components: relative hyperoxia and free radical stress. The first is the 90–95% hemoglobin oxygen saturation, which provides survival for extremely premature newborns during the early neonatal life, although these saturation values are not physiological for extremely premature newborns. This relative hyperoxia results in the slowdown of angiogenesis and organ development through oxygen-regulated gene expressions. This hyperoxic injury is not mediated by reactive oxygen species. The second type of oxygen toxicity, free radical injury, results in complex molecular changes. These alterations show parallelism with the severity of chronic morbidities in premature newborns, although, based on human studies, exogenous chemical antioxidants fail to provide prevention or effective therapy against oxygen toxicity. The positive relationship between free radical stress and prolonged lung damage can be well documented by biomarkers, the end-products of reactive oxygen species, such as carbonyl proteins, 8-oxyguanine, malondialdehyde, and many other oxidative derivatives of lipids, proteins, and nucleic acids measured by the methods of redoxomics [142]. A human study provides a new discovery that can be the link between free radical stress and BPD. Severe BPD can be characterized by the activation of the synthesis of oxygen free radicals; the process is strongly associated with the inflammatory response pathway. Preterm newborns with developing BPD present an increase in intermediate monocytes (CD14^++^CD16^+^), with the persistence of high levels of non-classical monocytes (CD14^+^ CD16^++^). Both monocyte subtypes are the main sources of TNF-alpha, interleukin-6, and interferon-alpha, leading to granulocyte activation and free radical production in cases of severe BPD. This study suggests future treatment strategies targeting monocytes instead of the administration of exogenous free radical scavengers [143]. Newborns have inducible, endogenous protective system that can express rapidly after reactive free radical stress. This consists of an enzymatic network; one of them is heme oxygenase-1, which can be upregulated by enzymatic inducers or gene transfer before the oxygen toxicity. These are probably more effective antioxidant strategies than chemical free radical chain breakers [144]. In chronic neonatal morbidities, other genes of antioxidant enzymes are the targets of therapeutic interventions, such as superoxide dismutase, catalase, glutathione-peroxidase, -reductase, -S-transferase, thioredoxin reductase 1, sulfiredoxin 1, and quinone oxidoreductase 1. These enzymes are elegantly summarized in an excellent review [145].

### 4.5. Antioxidants

LISA-nCPAP respiratory therapy together with the replacement of vascular growth factors should be considered as a basic intervention in neonatal care. In these optimal circumstances, the exogenous antioxidants might provide further favorable effects. The rationale for supplementation with exogenous antioxidants originates mainly from animal experiments. Vitamin E is the most frequently studied chain-breaking antioxidant in humans. Unfortunately, it does not provide preventive or therapeutic effects on neonatal chronic morbidities. The reevaluation of vitamin E is under consideration again since, today, neonatology enjoys the LISA-nCPAP therapy, prenatal steroid- and antibiotic prophylaxis [146]. N-acetylcysteine (NAC) might be a promising drug in the near future. The timing of the dosing may be the secret of its extreme efficacy in a well-planned randomized clinical trial, where NAC was given to mothers prenatally [147]. The rates of chronic neonatal morbidities decreased tremendously in the NAC-treated group compared to the control group, with NAC at 21% vs. placebo at 47%, relative risk of 0.45; 95% confidence interval (CI) 0.21–0.95. In the case of BPD, the preventive effect of NAC is more prominent, with NAC at 3% vs. placebo at 32%, with a relative risk of 0.10; 95% CI: 0.01–0.73. Premature newborns of NAC-treated mothers present a significantly higher plasma cysteine concentration without affecting the blood glutathione content. The study demonstrates a significant change in histone deacetylase expression; in this way, NAC treatment prevents the harmful epigenetic alterations in the placentas of women with preterm birth. The metabolism of amino acids with the -SH group is strictly linked to the production of hydrogen sulfide, a powerful, endogenous, antioxidant- and anti-inflammatory gas, which may be involved in this great beneficial phenomenon [148]. More basic- and clinical studies are needed to fully discover this complex preventive effect of NAC.

Melatonin is a strong antioxidant and free radical scavenger. A recent randomized control trial has shown that early melatonin administration in preterm newborns markedly reduces lipid peroxidation, suggesting that exogenous melatonin administration might be a potential strategy in the treatment of neonatal morbidities associated with oxidative stress [149]. 

Vitamin A, another lipid-soluble antioxidant, has been widely studied to prevent BPD, ROP, and IVH [150]. Vitamin A has remarkable antioxidant properties [151]. In VLBW infants, parenteral vitamin A supplementation slightly decreases the risk of chronic lung disease [152]. Others have found that early vitamin A supplementation might show good efficacy and safety in BPD prevention in premature infants [153]. However, enteral vitamin A administration does not reduce the severity of BPD [154,155]. Further studies of vitamin A are essential to evaluate its therapeutic potential in preterm infants.

Although parenteral lipid substitutes are not considered exogenous antioxidants, infused unsaturated fatty acids could compete with membrane-localized endogenous targets of reactive species. A nice observation supports this idea, lipid infusion prepared from fish oil prevents chronic neonatal morbidities compared to premature newborns who are treated by soybean-based lipid emulsion [156].

Today, the heme oxygenase–bilirubin system is one of the main focuses of vascular biology [157]. Heme oxygenase can behave as a protective enzyme, eliminate the toxic free heme, produce antioxidant bilirubin, and carbon monoxide, an anti-inflammatory gas at low concentrations [144,158]. In animal experiments, heme oxygenase delivered by mesenchymal stem cells to the broncho-alveolar system prevents hyperoxia mediated lung-, heart-, and kidney injuries. The study demonstrates a huge proinflammatory cytokine response for hyperoxia, but in the stem cell treated group, parallel to the amelioration of systemic multiple organic injury, a significant anti-inflammatory response is documented [159]. The lung histology presents dramatic improvements in pulmonary alveolarization and vasculogenesis, which emphasizes that the ultimate strategy in organ development is the vascular growth factor network [159]. Since the methodology of the measurement of free plasma indirect bilirubin has been published and there are many data about rapid ferritin changes after birth, clinical studies should be initiated on LISA-nCPAP intervention combined with the supplementation of growth factors, antioxidants, and antioxidant enzyme inducers in order to improve the morbidity-free survival rate in neonatal medicine [160,161].

## 5. Nephrogenesis and Kidney Function in Preterm Infants: The Lung–Kidney Interaction

### 5.1. Nephrogenesis in Preterm Newborns

Extremely premature infants are in a unique situation, since, in humans, de novo nephron formation continues until 36 weeks of gestation, with more than 60% of nephrons being formed in the last trimester of pregnancy [162]. Besides the negative effects on the kidney organogenesis of prematurity, any intrauterine or extrauterine stress can further result in a delay or stop in renal development. Neonates with lower birth weights, under 2,500 g, have significantly fewer glomeruli than those with a normal birth weight [163]. Therefore, nephron endowment arises from the complex interplay among genetic factors, perinatal events, and environmental exposures [164,165]. There is a long list of multiple pathologic molecular factors that have been implicated in reducing nephron endowment, including inflammatory signals, proinflammatory cytokines, reactive oxygen species, and antiangiogenic factors [166]. Renal function in premature newborns is capable of adaptation; the glomerular filtration rate (GFR) is relatively low in the preterm newborn after birth, and improves rapidly over the first week of life. The maturation of the renal function in preterm infants is a complex process. After birth, an intense increase in renal blood flow up to 15–18% of cardiac output can be observed by 6 weeks of life, paralleled with GFR increase. In extremely immature newborns, this process is further compromised by respiratory and systemic illnesses. Importantly, contrary to the severe inhibition of glomerular vascular development, the tubulopathy of prematurity is a transient, but clinically significant condition. The predominant feature of tubular immaturity is the decreased ability to reabsorb sodium through the nephron segment due to decreased transporter activities, Na^+^/K^+^ ATPase, Na^+^/H^+^ exchanger, and a limited responsiveness to aldosterone in the distal nephron segment. There are developmentally regulated changes in the relation between isoform composition and enzyme function of Na^+^,K^+^-ATPase [167]. Under physiologic conditions, rising glucocorticoid and thyreoid hormone levels in the immediate postnatal period induce a developmental increase in transport for most sodium-dependent transporters along the nephron [168]. Preterm kidneys have the limited ability to concentrate urine due to a poor osmotic gradient in the medulla, immaturity of the distal loop of Henle and diminished responsiveness of collecting tubules to ADH. This explains the necessity of a relatively high urine output in infants to excrete their solute load. It is speculated that the impaired renal concentration ability results in increased free water, which has been implicated in edema of prematurity, ventilator dependence, and the risk of developing BPD [169].

### 5.2. Acute Renal Injury: The Kidney–Lung Interplay in Premature Infants

A reduced nephron number, tubular immaturity, and decreased GFR in premature newborns increase the risk of acute renal injury (AKI), a condition that affects other organ functions.

There is a known and rather complex interplay between the cardiopulmonary system and the kidneys. For example, the severity of RDS and associated pulmonary vascular resistance affect the renin–angiotensin–aldosterone (RAAS) and atrial antidiuretic peptide (ANP) systems of the kidneys, thereby regulating salt and water excretion. A low systemic blood pressure and renal hypoperfusion, and the reduced peripheral vascular tone from catecholamine receptor insensitivity trigger RAAS activation. As a consequence of the reduced pulmonary venous return and high pulmonary vascular resistance (PVR), the more physiologic ANP system stimulation is blunted. The RAAS/ANP system plays a central role in the regulation of renal microcirculation to direct diuresis in premature infants [170]. Depending on the clinical situation and the variable presence of the above-mentioned factors, AKI may be present in either oliguric or non-oliguric forms.

The AWAKEN (assessment of worldwide acute kidney injury epidemiology in neonates) study retrospectively assessed a multinational, multicenter cohort of infants in 24 neonatal intensive care units (NICUs) and aimed to understand the epidemiology of AKI in neonates with the new unified definition. In this study, there was an overall incidence of 29.9% of AKI. In the preterm group, ELBW infants had a higher incidence of AKI (48%) compared to neonates born at 29–36 weeks gestation (28%). Additionally, any episode of AKI in neonates increased the risk for mortality three-fold compared to age-matched controls without an AKI [171]. Acute kidney injury (AKI), occurring in about 40% of extremely premature infants, is associated with both increased short-term morbidity and mortality and a greater long-term risk for chronic kidney disease (CKD). Although the precise molecular mechanisms are not fully understood, animal models have provided evidence for a deleterious impact of bidirectional kidney–lung injury [172,173,174,175,176]. Even non-oliguric AKI can lead to abnormal lung function and architecture [173]. In a patient cohort, 80% of infants who experienced AKI were born at <28 gestational age, whereas 73% of infants without documented AKI episodes were born after the 28th gestational week [177].

Non-oliguric presentation of AKI is common in premature infants, and the recognition of AKI may be challenging because the baseline creatinine value is often missing, diuresis data may be scarce, and there are only suboptimal AKI markers available [178]. AKI definitions are established on the basis of measuring parallel changes in serum creatinine and diuresis [171]. Apart from the characteristic acute diseases of premature newborns, pharmacokinetic and pharmacodynamic parameters differ, as the drug–excretion capacity is observed to be lower under the 33rd gestational week [179]. Biomarkers (CysC, NGAL, KIM-1, etc.) for the early detection of renal injury may significantly improve clinical practice in this patient population [166]. Although it was previously thought that most AKI was reversible, both experimental and clinical data indicate that recovery from AKI is often incomplete, with intrinsic forms of AKI causing irreversible damage, especially in premature newborns. VLBW infants represent a patient subpopulation already at risk of CKD because of low nephron number and endowment, with the presence of other risk factors [166,169,177].

Preterm birth interrupts the natural order of intrauterine development and inhibits the progression of organogenesis, leading to a reduced nephron endowment with simultaneous changes in other organ systems in the period of ”branching morphogenesis”, including the lung, heart, and vasculature. It was reported that neonates born between 29 and 32 weeks who develop AKI had a higher likelihood (four-fold higher odds) of moderate or severe BPD than those without AKI, and that difference remained after controlling for multiple factors [180].

While AKI episodes with either oliguric or non-oliguric presentation may be transient and self-limiting with questionable long-term renal consequences, episodes with more severe manifestation, especially when other acquired or inherited associated comorbidities are present, may occasionally require renal replacement therapy (RRT).

Unfortunately, RRT options in this population (<2500 g) are largely restricted, as many centers use peritoneal dialysis (PD) in the acute setting. However, some NICU centers have managed to perform CRRT using special devices even in LBW infants [181]. It needs to be emphasized that the management of AKI in this patient population is largely supportive. Neonatal AK is additionally associated with increased morbidity, specifically a longer length of stay, progressive chronic kidney disease (CKD), hypertension, and poor neurocognitive outcomes [182].

NIV is a benchmark in the management of several pathologies including RDS in premature infants. A prospective study found a greater occurrence of renal failure in patients ventilated by conventional strategies, compared to those treated with protective strategies such as NIV [183]. AKI may potentiate lung injury more prominently in this group due to the disruption of postnatal lung development and impaired angiogenesis leading to impaired alveolarization, suggesting the existence of both a special “kidney–lung organ crosstalk” and an interplay between AKI and BPD, two distinct inflammatory “multihit” processes that lead to substantial transcriptional changes in both organ systems [184].

## 6. Glucose Metabolism and Endocrine Characteristics of Premature Newborns Affecting LISA-nCPAP Efficacy

During fetal life, bioactive substances coming from the maternal circulation and produced by the placenta orchestrate fetal development in consonance with the developing fetal endocrine system. The external bioactive substances, growth factors, and hormones may reach the fetus via the umbilical cord or through the amniotic fluid. In cases of extremely premature births, a newborn misses all of these factors because they are synthesized or transferred actively or passively by the placenta to the fetus during the last trimester.

Early introduction of maternal milk feeding in NICUs and the proper management of nutritional needs for extremely immature infants are crucial. Exclusive breast milk feeding is recommended for infants during the first 6 months of life. Breast milk samples obtained from mothers delivering prematurely have higher protein, sodium, and certain hormone levels compared to milk from mothers who have delivered at term [185,186,187]. 

Since VLBW infants receiving breast milk often do not tolerate sufficient amounts of oral nutrients, beginning parenteral nutrition, including glucose, amino acids, and lipids, is highly recommended since premature infants use them not only for the anabolic state, but acquiring the energy needed for spontaneous breathing during LISA-nCPAP ventilation.

### 6.1. Glucose Metabolism in Premature Infants

During fetal life, as well as throughout the early neonatal period, glucose is the primary source of energy, especially for the brain. Due to their high brain/body ratio, VLBW infants utilize about three times more glucose per body kg compared to adults [188]. Continuous blood glucose monitoring (CGM) reveals hypoglycemia in 40% of extremely premature infants during the first two weeks of life, mainly within the first two postnatal days [189]. Hypoglycemia should be prevented by promoting breastfeeding as soon as possible after birth. Contrary to hypoglycemia, VLBW preterm infants, even with a 4–6 mg/kg/min parenteral glucose infusion, the basal need of glucose, can experience hyperglycemia [190]. In these cases, due to the disturbance of insulin synthesis, although proinsulin is produced, the insulin concentration is low. The biological activity of proinsulin is about one tenth of insulin. One main cause of this relative insulin deficiency is that the enteral uptake of glucose is a stronger insulin inducer compared to the effectivity of glucose provided parenteral route [191]. Another factor of relative insulin deficiency and hyperglycemia is the insufficient mass of adipose tissue compared to the skeletal and heart muscle, which limits the peripheral glucose uptake. Importantly, parenteral lipid preparations increase blood glucose concentration by 24% compared to baseline [192]. On the other hand, through the administration of amino acids in physiologically proper doses, blood glucose levels can be optimized. The explanation of this finding is that several amino acids, especially leucine, valine, isoleucine, glutamine, and arginine, enhance insulin secretion [193].

An important observation is that stress states in seriously ill neonates may lead to hyperglycemia by increasing the level of gluconeogenetic hormones, adrenaline, norepinephrine, and cortisol, with the opposing effects of insulin. In addition, exogenous hormone treatments, the most important ones being steroid drugs, applied in premature infants to ensure weaning from ventilator or treating BPD are also important pathological factors in the development of hyperglycemia [194]. Both hypo- and hyperglycemia require great attention, especially for extremely premature newborns with LISA-nCPAP ventilation, in order to provide enough physical energy for spontaneous breathing.

A study of 188 extremely-low-birth-weight (ELBW) infants with a gestational age of 27.1 ± 2.2 weeks and a birth weight of 814.9 ± 151.9 g demonstrates a 32.9% incidence of hyperglycemia, with 22.8% rate of insulin treatment. Subgroup analysis reveals that hyperglycemic infants have a smaller birth weight (*p* < 0.001), and suffer a higher incidence of severe ROP (*p* = 0.012) and mortality (*p* = 0.02). Logistic regression analysis shows that hyperglycemia is an independent risk factor for severe ROP (*p* < 0.001). In the rat model, neonatal hyperglycemia causes great vessel pathologies [195].

Amniotic insulin, a mirror of maternal blood insulin level, can reach the fetal gastrointestinal (GI) tract through swallowing, and it has an impact on intestinal maturation, improves feed tolerance, and influences microbiome development. Preterm birth interrupts these important fetal physiological processes, leading to disturbances in intestinal growth, cell maturation, and differentiation [196]. The first clinical trial where insulin (4 U/kg/day) was orally administered to preterm infants up to 28 days after delivery demonstrates better enteral milk tolerance and higher lactase activity [197]. More importantly, mothers’ milk contains a significantly higher concentration of insulin than cow’s milk, while insulin is not detectable in infant formulas [198].

### 6.2. Corticosteroid Treatment in Premature Newborns: Relation to LISA-nCPAP

Prenatal maternal steroid treatment has been proven to reduce infant mortality, the development of RDS, and the frequency of chronic morbidities [199]. For RDS prevention, direct, intramuscular betamethasone treatment can be given to fetuses in carefully selected cases [200]. Although prenatal steroid prophylaxis is extremely efficient in perinatal care, some clinical observations cannot be neglected. Prenatal steroid treatment affects the postnatal function of the hypothalamus–pituitary–adrenal axis. The acute suppression of cortisol synthesis is observed in the first week of life, but cortisol levels return to a normal range later in the first month, although a cortisol rise does not occur in response to pain stimulus. A similar phenomenon is present in mature, healthy newborns exposed to prenatal steroids [201]. Surprisingly, prenatal steroid treatment could be associated with mental problems in children, according to studies conducted at 8 and 16 years of age [202].

Early dexamethasone treatment in the first week of life promotes an earlier extubation of mechanically ventilated premature newborns, and reduces the risk of developing PDA and BPD. Great attention should be paid to potential side effects, since it may cause thrombotic phenomena, high blood pressure, gastrointestinal perforation, hyperglycemia, hypertrophic cardiomyopathy, and growth retardation. Since the overall beneficial effects of postnatal steroid treatment on survival rate and chronic morbidities are evident, its use is accepted in justified cases, but it must be administered for the shortest possible period of time and at the lowest effective dose [203].

### 6.3. Thyroid Function: Hormone Substitution

During fetal life, starting at the 12th postconceptional week, the thyroid gland produces thyroxine (T4) and triiodothyronine. The secretion increases with advancing gestational age.

In the cases of extremely premature newborns, hypothyroidism is frequently observed due to the immaturity of the hypothalamic–pituitary–thyroid axis. The main pathologies of neonatal hypothyroidism are neurocognitive delay and cholestasis [204]. Moreover, thyroid functions influence postnatal adaptation; maternal TRH treatment enhances surfactant production in the fetal lung [205]. Thyroid hormones are present not only in the maternal and fetal plasma, but also in the amniotic fluid [206]. TSH and thyroxine are present in the breast milk produced for term or preterm infants. The two groups had similar levels of TSH, but term milk contains higher amounts of thyroxine (11,245.5 ± 73.8 vs. 671.6 ± 61.2 nmol/L) during the first 6 months of lactation [207]. Pasteurization decreases the TSH and thyroxine concentrations by 73.8- and 22.4%, respectively. Holder pasteurization is known to influence the levels of bioactive factors and compounds in human milk [208,209], but the TSH content is not affected, contrary to pasteurization (Table 3) [210].

Recently, an important study demonstrated the beneficial effects of early thyroxine supplementation in preterm infants. It improves neurodevelopment scores at the age of 3–4 years in infants born below 28 weeks gestation [211]. This observation highlights the need for studies investigating early supplementation, especially when VLBW infants are fed with donor milk or formula.

It is evident that nutrition, together with well-controlled hormone substitution, serves as one of the basic foundations of the success of LISA-nCPAP therapy. Importantly, the relationship between feeding and NIV is mutual. The introduction of LISA-nCPAP helps advance enteral feeding. Higher amounts of enteral feeding are tolerated at the end of the first week of life in NIV patients compared to the MV group. Of the premature newborns, 55.9% regained birth weight by the end of the first week of life in LISA-nCPAP group, compared to 32.0% in the control group (*p* < 0.001) [212].

## 7. Inflammatory Characteristics in Extremely Immature Newborns

Premature infants are at risk of chronic inflammation. Premature labor interrupts the maternal influence on immune regulation, e.g., through placental circulation or bioactive molecules of the amniotic fluid. Breast milk provides primary metabolic fuels, hormones, vitamins, microorganisms, immunologically active cells and molecules to promote the well-being of the infant, and also controls immune functioning in the early postnatal life. These effects of breastfeeding ensure proper physical and mental development in the offspring.

### 7.1. Postpartum Immune Response Is Balanced by Breast Milk

Birth marks a profound and rapid rearrangement of the immune responses, as the newborn enters a potentially harmful, pathogen-rich environment and is exposed to novel antigens [213]. This transition requires effective immune recognition and neutralization of pathogenic organisms and the immune tolerance of food antigens, the microbiome, and neoantigens of developing tissues [213,214,215]. 

Any imbalance in these two tasks may lead to a destructive immune response and inflammation, or, on the other hand, the immune evasion of pathogens or malfunctional cells, self-immunity, or allergy. Indeed, a preterm infant is at risk of such an immune imbalance [216,217]. Preterm infancy is associated with the immaturity of immune mechanisms, increasing vulnerability to infections, which is a main cause of perinatal fatalities in the case of preterm delivery [218]. Intrauterine infection also exacerbates inflammation in the fetus, causing premature delivery and a high risk of perinatal mortality [216]. Premature infants also have a high risk of neonatal sepsis and may have an exacerbated systemic inflammation and “hyper-reactive” innate immune responses [219]. This may be due to the lack of immune control mechanisms, such as cytokines, that limit T-cell activation [216]. Signal mechanisms that orchestrate the immune functioning after birth are thus vital for the survival of a preterm infant. 

In a term infant, breast milk plays a central role in the balance of postpartum immune functioning [220]. Breast milk contains a complex matrix of effector immune cells, Th1 cytokines, and immunoglobulins that allow for a passive immunization of the newborn against several pathogens. The breast milk also promotes the establishment of the gut microbiome, and contains anti-inflammatory cells, molecules, and enzymes that protect the infant from an uncontrolled inflammation and destructive immune response [221,222,223]. Relevant anti-inflammatory mediators of the colostrum and breast milk include long-chain polyunsaturated fatty acids, prostaglandins, prostacyclin, anti-proteases, antioxidants, Th2 cytokines, lactose-derived 2′-fucosyllactose and 6′-sialyllactose, a wide range of microRNA species, and PAF-acetylhydrolase. Most of these molecules are lacking in formula milk [220,224,225]. Accordingly, the lack of breast feeding is associated with the increased risk of immune pathologies in infants and children, such as type 1 diabetes, premature loss of thermogenic adipose tissue, infection-triggered autoimmunity, allergy, and especially in extremely premature infants, chronic morbidities, BPD, ROP, IVH, and NEC [222,225,226]. Infants receiving LISA may be fed with breast milk, reducing the risk for uncontrolled chronic inflammation. 

### 7.2. Immune-Regulating Molecules of Breast Milk

Docosahexaenoic acid (DHA) is a long-chain polyunsaturated fatty acid that exerts anti-inflammatory potential [227,228,229]. Impaired DHA synthesis leads to a “hyper-reactive“ pro-inflammatory macrophage phenotype, which may cause tissue damage [227]. Further, anti-inflammatory lipids of breast milk include prostaglandin E2, prostaglandin F2 alpha, and prostacyclin. These mediators are secreted by the mammary gland cells and the immune cells of the breast milk. Prostaglandin E2 secretion is, for instance, associated with breast milk macrophages [230]. Eventually, these lipid mediators are lacking in cow-milk-based formula [231]. Similarly, lactose-derived milk oligosaccharides–2′-fucosyllactose and 6′-sialyllactose have been shown to inhibit Toll-like receptor 4 (TLR4), a pathogen recognition receptor and a central stimulator of pro-inflammatory cytokine expression. Supplementation of formula milk with 2′-fucosyllactose and 6′-sialyllactose effectively reduced the NEC in animal studies [223]. 

The enzymes of breast milk also protect from uncontrolled inflammation. Platelet-activating factor (PAF) is one of the most proinflammatory mediators, and is present in the colostrum and breast milk [232]; however, PAF is responsible for the exacerbation of NEC in neonates. Breast milk contains a PAF-degrading enzyme, so-called PAF-acetylhydrolase (PAF-AH), also known as phospholipase A2 group 7 (PLA2G7, EC 3.1.1.47). PAF-AH activity is associated with the aqueous phase of breast milk [233]. PAF-AH protects the infant from an excess PAF level, and hence, breast milk feeding in preterm infants—a vulnerable group with increased risk for chronic morbidities—may protect from a tissue-damaging inflammation. PAF-AH activity decreases with advancing lactational age and is sensitive to gestational age [234]. Deficiency in PAF-AH worsens inflammation in animal studies [235], while milk supplementation with PAF-AH appears protective [236]. Macrophages may be additional sources of PAF-AH in breast milk, and their PAF-AH secretion is dependent on functional very-low-density lipoprotein receptors. Consistently, mice lacking very-low-density lipoprotein receptors produce milk defective in PAF-AH, and the offspring suffer from tissue-damaging inflammation and growth retardation [236]. Of note, both mouse and human macrophages express PAF-AH, allowing for the safe elimination of excess PAF [225]. 

The colostrum and breast milk supply cytokines to the newborn to establish an adequate balance of Th1 and Th2 responses [237]. Breast milk polarizes murine macrophages toward an anti-inflammatory M2 state [238], and contains Th2 cytokines [239,240]. These cytokines may protect the gastrointestinal mucosa, aid the establishment of immune tolerance towards the microbiome and food antigens. Antagonism of Toll-like receptor (TLR) signaling is a key effect of breast milk [224], and this effect is plausibly augmented by a wide range of microRNA species [241], that are delivered in microvesicles of the breast milk [242]. 

## 8. Rediscovering Old Drugs

The use of rediscovered NIV techniques in everyday practice of neonatal intensive care in the surfactant era has led to the rediscovery of old drugs as well, such as caffeine, steroids, non-steroid anti-inflammatory drugs, and mother’s milk. In a single-center study, the rate of BPD decreased by more than 50% after starting NIV ventilation together with the early use of caffeine [243]. Novel research reveals that there are other beneficial effects of caffeine besides preventing or treating apnea in prematurity. Caffeine, as an adenosine A2A receptor antagonist, inhibits oxidative stress and apoptosis and promotes the proliferation of alveolar epithelial cells; these observations raise the hypothesis that other A2A receptor antagonists could serve as preventive and therapeutic agents for BPD [244].

Steroids in neonatal medicine should be appropriately used with caution, since the 36-week gestational age is not the best measure for long-term adverse outcomes. In order to decrease the rate of BPD, steroid treatment should be started after 7 days of life. This primary goal can be reached, but neurodevelopmental morbidities and chronic obstructive pulmonary disease may occur [245,246]. Until now, there are not enough data on inhaled corticosteroids to prevent BPD, so local administration is not justified as a steroid alternative route [247]. The overall conclusion is supportive of steroid use after the age of 7 days in premature newborns to improve BPD-free survival, but with important remarks. Systemic administration is justified if the patient cannot be weaned from the ventilator. Further long-term follow-up should be carried out in order to gain objective data about cognitive, executive brain function, academic performance, behavior, mental health, motor function, and lung function [247]. Inhaled steroids might be beneficial for preterm infants with respiratory symptoms. In a randomized clinical trial on premature infants at a mean gestational age of 28 weeks and a postnatal age of 10.5 months, it has been shown that late coughing and wheezing reduced by 37% in response to steroid inhalation compared to the placebo group in the first year of life [248]. Although the symptoms of this chronic lung morbidity, BPD, presents the symptoms of asthma, it is mainly the consequence of bronchial hyperreactivity. 

Inflammation has been a central issue in the chronic morbidities of immature newborns in neonatal medicine. Since respiratory support represents the main intervention for this sensitive population and, importantly, inflammatory reactions have a multicausative nature, one can conclude that besides bacterial, viral, and protozoal infective organisms, the mechanical stress of respiratory support could lead to inflammation. The situation is more complex, since reactive oxygen species, hyperoxia, certain drugs, and endogenous non-infective mediators, such as free heme, hemoglobin, and lipid peroxidation end-products, could play synergistic roles in systemic inflammatory response. Our focus is the LISA-nCPAP intervention, where avoiding the baro-, volutrauma, and airway touching stress, these proinflammatory factors are prevented. Importantly, in the case of the LISA technique, intubation is not allowed, even though the injury of the airways does not happen. 

The introduction of NIV techniques improves the outcome of extremely premature newborns [249]. In an elegant study, in a hyperoxic cell death model, inflammatory reactions, inflammasome activation, caspase-1 activation, interleukin-1beta (IL-1) and interleukin-18 induction can be observed in the brain tissue. The targets of the high oxygen concentration are the nuclear factor erythroid 2-related factor 2 (Nrf2) and Kelch-like ECH-associated protein-1 (Keap1), and the signal transduction pathway uses the nuclear factor kappa-light chain enhancer of the activated B-cell (NF-kappaB) system, both in the lungs and brain [132,250]. Antibiotics used for the treatment of sepsis or the eradication of colonization present controversial consequences for chronic neonatal complications. Prolonged administration of antibiotics right after birth, the so-called prolonged early use, is associated with the development of BPD, and increases the incidence of necrotizing enterocolitis. The only indication for neonatal antibiotic use remains infection and sepsis [251]. Based on a novel multicenter prospective study, antibiotic overexposure triples the risk of BPD [252]. After exposure of human bronchial epithelioid cells to mechanical stretch, IL-13, metalloprotein-9, and transient receptor potential canonical 1 (TRCP1) are significantly increased within half an hour of the initiation of stretch stress [253]. This study underlines the importance of the LISA-nCPAP technique among NIV strategies, which means avoiding intubation to prevent the short-term mechanical stretch and its inflammatory consequences.

A mother’s colostrum and milk decrease the incidence of late onset sepsis in premature newborns, which emphasizes the role of nutrition in immune function and chronic morbidities in premature newborns. Consequently, mother’s milk has a distinct importance in neonatal medicine [254].

## 9. Conclusions

New discoveries of NIV methods and adjuvant therapies for supporting fetal–neonatal organ developments in perinatal–neonatal medicine result in a great improvement in the chronic morbidity-free survival rate of extremely premature newborns. Although the LISA-nCPAP ventilatory strategy and new adjuvant interventions are independently able to give this population a chance to live a healthy adult life, more importantly, the combination of them may synergistically help the realization of a positive future. 

**Figure 1:**
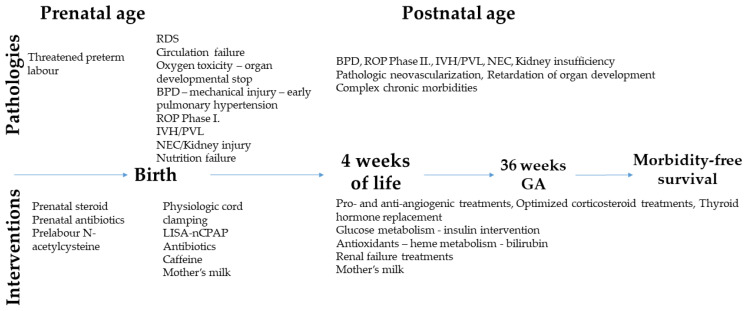
Prevention of chronic morbidities of extremely premature newborns by LISA-nCPAP respiratory therapy and adjuvant perinatal strategies.

New NIV interventions are emerging tools in order to improve the chronic morbidity-free survival rate in extremely premature newborns, and the same is true for new adjuvant therapies, as well (Figure 1). Non-invasive positive pressure ventilation (nIPPV) is increasingly used to avoid invasive ventilation in preterm infants [255,256]. A recent Cochrane review reported that nIPPV is superior to nCPAP for decreasing respiratory failure and the need for mechanical ventilation in preterm infants with RDS [257]. So far only a few trials have compared the use of nCPAP and nIPPV as the initial mode of respiratory support before LISA. In their prospective cohort study, Szczapa et al. reported that LISA with nIPPV was not superior over nCPAP in terms of the need for invasive ventilation [29]. A randomized controlled trial demonstrated that nIPPV within the LISA approach reduced the rate of mechanical ventilation within the first 72 h of life in infants born at 26–32 weeks gestation [258]. However, this reduction was not found in the subgroup of infants born at less than 30 weeks gestation. The NIV-MISA-RDS trial (NCT05137340) will assess whether the use of nCPAP or nIPPV as primary support before LISA is associated with lower non-invasive nasal respiratory support failure rates in preterm infants born between 24 and 29 weeks gestation [259].

The preventive and treatment strategies should be adjusted to certain time frame patterns, and all of the components of the pathophysiology should be taken into account to reach the best rate of chronic morbidity free survival. LISA-nCPAP ventilatory support is one of the most important preventive tools for chronic morbidity free survival in extremely premature infants, it deserves new adjuvant strategies during the neonatal intensive care.

## Figures and Tables

**Table 1:** Randomized controlled trials comparing LISA with the continuation of CPAP or S-ETT.

Reference	Study Design	Intervention	Comparator	Main Results
LISA vs. continuation of CPAP				
Gopel et al., 2011 (AMV trial) [34]	12 centers 26–28 weeks GA Various types of surfactant (poractant alfa, beractant, bovactant) Surfactant dose: 100 mg/kg Analgosedation: per physician decision	FiO_2_ > 0.3, CPAP ≥ 4 cmH_2_O Cologne method 4 Fr NGT LISA was allowed to be repeated if FiO_2_ > 0.4 Rescue intubation according to the judgment of physician (unclear tresholds)	Rescue intubation and surfactant according to the judgement of the attending physician (unclear tresholds) Extubation was recommended *	*n* = 220, LISA group *n* = 108 (65 LISA, 15 S-ETT per physician decision, 28 never received surfactant), comparator group *n* = 112 (72 S-ETT, 1 LISA per physician decision, 39 never received surfactant) ↓ need for MV on day 2 or 3 (28% vs. 46%, *p* = 0.008) ↓ need for MV during hospital stay (33% vs. 73%, *p* < 0.0001) ↓ median days on MV (0 vs. 2 days, *p* < 0.0001) ↓ need for O_2_ at 28 days (30% vs. 45%, *p* = 0.032)
Dargaville et al., 2021 (OPTIMIST-A trial) [37]	33 centers 25–28 weeks GA Double-blinded Surfactant criteria: FiO_2_ > 0.3, CPAP 5–8 cmH_2_O Surfactant: poractant alfa Surfactant dose: 200 mg/kg Premedication: atropin and sucrose per physician decision	Hobart method 16G vascular catheter or LISAcath LISA was not allowed to be repeated Rescue intubation if FiO_2_ ≥ 0.45 (or 0.4 per physician decision) or recurrent apnea or persistent respiratory acidosis	Sham treatment (only transient repositioning) Rescue intubation if FiO_2_ ≥ 0.45 (or 0.4 per physician decision) or recurrent apnea or persistent respiratory acidosis After intubation, surfactant could be administered according to clinical judgement.	*n* = 485 ↔ death or BPD (43.6% vs. 49.6% *p* = 0.1) ↓ death (10% vs. 7.8%, *p* = 0.51) ↓ BPD in survivors (37.3% vs. 45.3%, *p* = 0.03) ↓ PTX (4.6% vs. 10.2%, *p* = 0.005) ↓ CPAP failure (36.5% vs. 72.1%, *p* < 0.001)
LISA vs. surfactant administration via ETT with extubation				
Kribs et al., 2015 (NINSAPP trial) [35]	13 centers 23–26 weeks GA Surfactant criteria: FiO_2_ > 0.3, CPAP cmH_2_O or Silverman score ≥ 5 Surfactant type: poractant alfa Surfactant dose: 100 mg/kg No premedication	Cologne method 4 Fr NGT	S-ETT then MV as per local standards Extubation criteria: FiO_2_ > 0.3 and MAP < 10 cmH_2_O	*n* = 211 ↔ survival without BPD (67.3% vs. 58.7%, *p* = 0.2) ↓ need for MV (74.8% vs. 99% ^∆^, *p* = 0.04) ↓ median duration of MV (5 days vs. 7 days, *p* = 0.031) ↓ PTX (4.8% vs. 12.6%, *p* = 0.04) ↓ IVH (10.3% vs. 22.1%, *p* = 0.02) ↑ survival without major complications (50.5% vs. 35.6%, *p* = 0.02).
Olivier et al., 2017 [36]	3 centers 32–36 weeks GA Surfactant type: beractant Surfactant dose: 100 mg/kg Fentanyl 1 µg/kg + atropin 20 µg/kg	FiO_2_ > 0.35, CPAP 6 cmH_2_O Cologne method 5 Fr NGT	Rescue intubation and surfactant according to the judgment of the attending physician (unclear tresholds), Extubation criteria was not reported (extubation was not routinely performed)	*n* = 45 ↓ primary outcome (need for MV or respiratory failure criteria or PTX requiring chest drain) (33% vs. 90%, *p* ≤ 0.001)
LISA vs. INSURE				
Kanmaz et al., 2012 (Take Care study) [38]	Single center <32 weeks GA Surrfactant criteria: FiO_2_ ≥ 0.4, CPAP 5–7 cmH_2_O Surfactant type: poractant alfa Surfactant dose: 100 mg/kg No premedication	Take Care method 5 Fr NGT (1 bolus in 30–60 s)	Double-lumen ETT During surfactant instillation (30 s), 20/5 cmH_2_O pressure PPV was performed with a T-piece device, then extubation to CPAP	*n* = 200 ↓ CPAP failure (30% vs. 45%, *p* = 0.02) ↓ mean duration of CPAP (78 h vs. 116 h, *p* = 0.002) ↓ mean duration of MV (35.6 h vs. 64.1 h, *p* = 0.006) ↓ BPD (10.3% vs. 20.2%, *p* = 0.005)
Mirnia et al., 2013 [39]	3 centers 27–32 weeks GA Surfactant criteria: FiO_2_ ≥ 0.3, CPAP 8–10 cmH_2_O Surfactant type: poractant alfa Surfactant dose: 100 mg/kg Atropin 5 µg/kg	Take Care method 5 Fr NGT (1 bolus in 1–3 min)	No detail reported	*n* = 136 ↔ CPAP failure (19% vs. 22%, *p* = 0.6) ↓ mortality (3% vs. 15.7%, *p* = 0.01)
Mohammadizadeh et al., 2015 [40]	2 centers ≤34 weeks GA and and BW 1000–1800 g Surfactant criteria: FiO_2_ > 0.3, CPAP 6 cmH_2_O and/or Silverman score >4 Surfactant type: poractant alfa Surfactant dose: 200 mg/kg Atropin 25 µg/kg	Cologne method 4 Fr NGT	2.5–3.0 ETT Bolus injection then PPV with a T-piece device for at least 1 min or until SpO_2_ ≥87%, then extubation to CPAP	*n* = 38 ↔ CPAP failure (15.8% vs. 10.5%, *p* = 0.99) ↓ duration of O_2_ therapy (243.7 h vs. 476.8 h, *p* = 0.018) ↓ adverse events during surfactant administration (31.6% vs. 63.2%, *p* = 0.049)
Bao et al., 2015 [41]	Single center 28–32 weeks GA Surfactant criteria: FiO_2_ > 0.3 for 28–29 weeks GA, FiO_2_ > 0.35 for 30–32 weeks GA, CPAP 7–8 cmH_2_O Surfactant type: poractant alfa Surfactant dose: 200 mg/kg No premedication	Hobart method 16G vascular catheter (5 boluses in 3–5 min)	Surfactant injection in 2–3 boluses in 3 min, brief MV (details not reported), then extubation to CPAP	*n* = 90 ↔ CPAP failure (17% vs. 23.3%, *p* = 0.44) ↓ duration of MV + CPAP (13.2 days vs. 15.9 days, *p* = 0.03)
Li et al., 2016 [42]	Single center 27–31 weeks GA Surfactant criteria: RDS grade I-II on CXR Surfactant type: poractant alfa Surfactant dose: various doses No premedication	Cologne method	No detail reported	*n* = 40 Both LISA and INSURE caused a transient impairment in cerebral autoregulation, the duration of this effect was shorter in the LISA group (<5 min vs. 5–10 min)
Mosayabi et al., 2017 [43]	Single center 28–34 weeks GA Surfactant criteria: FiO_2_ > 0.4, CPAP 5–8 cmH_2_O Surfactant type: poractant alfa Surfactant dose: 200 mg/kg No premedication	Take Care method 5 Fr NGT	Surfactant injection in 1–3 min, manual ventilation (bagging), then extubation to CPAP 3 min	*n* = 53 ↔ CPAP failure (38.3% vs. 36.8%, *p* = 0.827)
Choupani et al., 2018 [44]	Single center GA or BW criteria not reported Surfactant criteria: FiO_2_ > 0.4, CPAP 6 cmH_2_O Surfactant type: poractant alfa Surfactant dose: 200 mg/kg No premedication	Hobart method 16G vascular catheter (small aliquots in 2–4 min)	Bolus injection, then PPV with a T-piece device for at least 1 min or until SpO_2_ ≥87%, then extubation to CPAP	*n* = 104 ↔ CPAP failure (15.4% vs. 25%, *p* = 0.222) ↓ incidence of hypoxia (SpO_2_ < 80%) during surfactant administration (11.5% vs. 28.8%, *p* = 0.028)
Halim et al., 2019 [45]	Single center ≤34 weeks GA Surfactant criteria: FiO_2_ > 0.4, CPAP 5–7 cmH_2_O Surfactant type: beractant Surfactant dose: 100 mg/kg No premedication	Take Care method 6 Fr NGT	Bolus injection, then PPV with a T-piece device for 15–20 min, then extubation to CPAP	*n* = 100 ↓ need for MV at any time (30% vs. 60%, *p* = 0.003) ↓ median duration of MV (40 h vs. 71 h, *p* = 0.004)
Boskadabi et al., 2019 [46]	Single center <32 weeks GA and and BW <1500 g Surfactant criteria: FiO_2_ > 0.4, CPAP 5–8 cmH_2_O Surfactant type: poractant alfa Surfactant dose: 200 mg/kg No premedication	Take Care method 5 Fr NGT	Bolus injection, bagging for 30–60 s, then extubation to CPAP	*n* = 40 ↓ CPAP failure (0% vs. 30%, *p* = 0.002)
Jena et al., 2019 [47]	3 centers ≤34 weeks GA Surfactant criteria: FiO_2_ > 0.3, CPAP 6 cmH_2_O Surfactant type: bovine lipid extract surfactant suspension Surfactant dose: 135 mg/kg No premedication	Hobart method 16G vascular catheter or Take Care method 6 Fr NGT based on individual preference	Bolus injection, then PPV with a T-piece device (no detail reported), then extubation to CPAP	*n* = 350 ↓ CPAP failure (19% vs. 40%, *p* < 0.01) ↓ duration of CPAP (4 days vs. 8 days, *p* < 0.01) ↓ duration of O_2_ therapy (6 days vs. 12 days, *p* < 0.01) ↓ BPD (3% vs. 17%, *p* < 0.01)
Yang et al., 2020 [48]	Single center 32–36 weeks GA Surfactant criteria: FiO_2_ > 0.4, CPAP 6 cmH_2_O Surfactant type: poractant alfa Surfactant dose: 200 mg/kg No premedication	Cologne method 4 Fr NGT	Bolus injection then PPV (no detail reported), then extubation to CPAP	*n* = 97 ↔ need for MV (8.5% vs. 6%, *p* = 0.8) ↔ duration of MV (3.1 days vs. 3.3 days, *p* = 0.27)
Han et al., 2020 [49]	8 centers 25–31 weeks GA Surfactant criteria: FiO_2_ > 0.4, CPAP 6–8 cmH_2_O Surfactant type: calf pulmonary surfactant preparation Surfactant dose: 70–100 mg/kg No premedication	Modified Cologne method with 10 cm ophthalmic forceps 4 Fr NGT (in mini boluses over 1–5 min)	Bolus surfactant, MV as per local standards, then extubation if FiO_2_ < 0.3 and MAP < 8 cmH_2_O	*n* = 298 ↔ BPD (19.2% vs. 25.9%, *p* = 0.17) ↓ PDA (41.1% vs. 60.5%, *p* = 0.001) Subgroup analysis of <30 weeks GA (*n* = 51): ↓ BPD (29% vs. 70%, *p* = 0.004)
Gupta et al., 2020 [50]	Single center 28–34 weeks GA Surfactant criteria: FiO_2_ > 0.3; NIPPV fr 40/min, PIP 12–15 cmH_2_O, PEEP 5–6 cmH_2_O Surfactant type: poractant alfa Surfactant doze: 200 mg/kg No premedication	Cologne method 5 Fr NGT (1 mL aliquots, each lasting for 10 s)	Bolus injection, bagging for 30–60 s, then extubation to NIPPV	*n* = 58 ↔ CPAP failure (10.34% vs. 20.69%, *p* = 0.47)
Pareek et al., 2021 [51]	Single center 28–36 weeks GA Surfactant criteria: NIPPV (unclear tresholds) at least 2 of the following criteria: Silverman score ≥ 4 or FiO_2_ > 0.3 for <30 weeks GA and FiO_2_ > 0.4 for ≥30 weeks GA or > stage II RDS on CXR Surfactant type: not reported Surfactant dose: 100 mg/kg No premedication	Cologne or Take Care method based on individual preference 5 Fr NGT	Bolus injection, then PPV with a T-piece device (no detail reported), then extubation to the NIPPV	*n* = 40 ↔ CPAP failure (30% vs. 30%, *p* = 0.99)
Anand et al., 2022 [52]	Single center 26–34 weeks GA Surfactant criteria: FiO_2_ > 0.3 within 6 h of life Surfactant type: beractant Surfactant dose: 100 mg/kg No premedication	Take Care method 8 Fr NGT	Injection in four equal aliquots, bagging between aliquots, then extubation to CPAP	*n* = 150 ↔ duration of respiratory support (120 h vs. 120 h *p* = 0.618) ↓ need for MV (9.5% vs. 25%, *p* = 0.017) ↓CPAP failure (17.5% vs. 38.1%, *p* = 0.005)

Table notes: First % values refer to the LISA group and second values refer to the comparator group. ↑—significantly higher, ↓—siginificantly lower, ↔—not significantly different, * Despite the recommendation, of 81 infants who were intubated on the first day after birth, only 27 (33%) were extubated within the first 24 h, ^∆^ One infant was not intubated since FiO_2_ dropped below the treatment treshold immediatley after randomization Cologne method—insertion of a flexible catheter with Magill’s forceps, Take Care method—insertion of a flexible catheter without Magill’s forceps, Hobart method—insertion of a semi-rigid catheter. Surfactant administration via ETT with extubation in these trials, control infants remained intubated after surfactant delivery, with extubation after a period of mechanical ventilation. Abbreviations: LISA—less invasive surfactant administration, S-ETT—surfactant administration via endotracheal tube with (INSURE) or without extubation, GA—gestational age, NGT—nasogastric tube, FiO_2_—fraction of inspired oxygen, MV—mechanical ventilation, INSURE—intubation–surfactant–extubation, BPD—bronchopulmonary dysplasia, IVH—intraventricular hemorrhage, CPAP—continuous positive airway pressure, CPAP failure—need for mechanical ventilation within 72 h of birth, RDS—respiratory distress syndrome, CXR—chest X-ray, ETT—endotracheal tube, PPV—positive pressure ventilation, PTX—pneumothorax, BW—birth weight, PDA—patent ductus arteriosus, NIPPV—non-invasive positive pressure ventilation.

**Table 2:** Meta-analyses comparing LISA with S-ETT.

Reference	Study Description	Results
Isayama et al., 2016 [53]	Network meta-analysis 30 RCTs, *n* = 5598 4 LISA studies, *n* = 637 1 LISA vs. MV 3 LISA vs. INSURE	Compared to MV, LISA had the lower odds of death or BPD (OR 0.49, 95% CrI 0.3–0.79; absolute RD 164 fewer per 1000 infants; 95% CrI 57–253 fewer per 1000 infants, moderate quality of evidence) BPD (OR 0.53, 95% CrI 0.27–0.96; absolute RD 133 fewer per 1000 infants; 95% CrI 9–234 fewer per 1000 infants, moderate quality of evidence) severe IVH (OR 0.44, 95% CrI 0.19–0.99; absolute RD 58 fewer per 1000 infants; 95% CrI 1–86 fewer per 1000 infants, moderate quality of evidence) Compared to CPAP alone, LISA had the lower odds of death or BPD (OR 0.58, 95% CrI 0.35–0.93; absolute RD 112 fewer per 1000 infants; 95% CrI 16–190 fewer per 1000 infants, moderate quality of evidence) air leak (OR 0.24, 95% CrI 0.05–0.96; absolute RD 47 fewer per 1000 infants; 95% CrI 2–59 fewer per 1000 infants, very low quality of evidence) Ranking probabilities indicated that LISA was the best strategy (surface under the curve 0.85–0.94).
Rigo et al., 2016 [54]	6 RCTs, *n* = 895 2 LISA vs. MV + surfactant 4 LISA vs. INSURE	Compared to S-ETT, LISA resulted in decreased risk of BPD (RR = 0.71, 95% CI 0.52–0.99; NNB = 21) death or BPD (RR = 0.74, 95% CI 0.58–0.94; NNB = 15) early CPAP failure (RR = 0.67, 95% CI 0.53–0.84; NNB = 8) need for MV during NICU stay (RR = 0.69, 95% CI 0.53–0.88; NNB = 6) Compared to INSURE, LISA decreased the risk of BPD or death (RR = 0.63, 95% CI 0.44–0.92; NNB = 11) early CPAP failure (RR = 0.71, 95% CI 0.53–0.96; NNB = 11)
Aldana-Aguirre et al., 2017 [10]	6 RCTs, *n* = 895 2 LISA vs. MV + surfactant 4 LISA vs. INSURE	Compared to S-ETT, LISA reduced the risk of death or BPD (RR = 0.75, 95% CI 0.59–0.94) BPD among survivors (RR = 0.72, 95% CI 0.53–0.97) CPAP failure (RR = 0.71, 95% CI 0.53–0.96) need for MV during NICU stay (RR = 0.66, 95% CI 0.47–0.93) and LISA lead to an increased risk of surfactant reflux (RR = 2.52, 95% CI 1.47–4.31)
Barkhuff et al., 2019 [55]	7 RCTs, *n* = 895 (*n* = 940 for PTX) 3 LISA vs. MV + surfactant 4 LISA vs. INSURE	Compared to S-ETT, LISA resulted in a lower risk of death or BPD (RR = 0.74, 95% CI 0.59–0.94; NNB = 14) PTX (RR = 0.61, 95% CI 0.37–1) CPAP failure (RR = 0.74, 95% CI 0.65–0.85) Compared to INSURE, LISA decreased the risk of death or BPD (RR = 0.66, 95% CI 0.46–0.93; NNB = 11) CPAP failure (RR = 0.72, 95% CI 0.53–0.97)
Abdel-Latif et al., 2021 [56]	16 RCTs (*n* = 2164) 10 studies (*n* = 1324) for death or BPD 12 studies (*n* = 1422) for CPAP failure 5 studies (*n* = 857) for severe IVH 11 studies (*n* = 1424) for death during first hospitalization 11 studies (*n* = 1567) for BPD among survivors	Compared to S-ETT, LISA was associated with a lower risk of death or BPD (RR = 0.59, 95% CI 0.48–0.73; NNB = 9; moderate quality of evidence) CPAP failure (RR = 0.63, 95% CI 0.54–0.74; NNB = 8; moderate quality of evidence) severe IVH (RR = 0.63, 95% CI 0.42–0.96; NNB = 22; low quality of evidence) death during first hospitalization (RR = 0.63, 95% CI 0.47–0.84; NNB = 20; low quality of evidence) BPD among survivors (RR = 0.57, 95% CI 0.45–0.74; NNB = 13; moderate quality of evidence) Compared to INSURE, LISA decreased the risk of death or BPD (RR = 0.52, 95% CI 0.4–0.68; NNB = 9) death during first hospitalization (RR = 0.6, 95% CI 0.44–0.82; NNB = 19) BPD among survivors (RR = 0.57, 95% CI 0.44–0.75; NNB = 14) CPAP failure (or not intubated, but reaching the failure criteria) (RR = 0.72, 95% CI 0.53–0.96; NNB 11) need for MV at any time (RR = 0.7, 95% CI 0.54–0.9; NNB = 7)
Bellos et al., 2021 [57]	Network meta-analysis 16 RCTs and 20 observational studies (*n* = 13,234)	Compared with INSURE, LISA lowered the rates ofmortality (OR = 0.64, 95% CI 0.54–0.76; moderate quality of evidence) MV (OR = 0.43, 95% CI 0.29–0.63; moderate quality of evidence) BPD (OR = 0.57, 95% CI 0.44–0.73; moderate quality of evidence) PVL (OR = 0.66, 95% CI 0.53–0.82; moderate quality of evidence) NEC (OR = 0.67, 95% CI 0.41–0.9; low quality of evidence) In RCTs, LISA decreased the rates of MV at any time (OR: 0.39, 95% CI: 0.26 to 0.60), but not the incidence of the remaining outcomes.

Abbreviations: LISA—less invasive surfactant administration, S-ETT—surfactant administration via endotracheal tube with (INSURE) or without extubation, MV—mechanical ventilation, INSURE—intubation–surfactant–extubation, BPD—bronchopulmonary dysplasia, OR—odds ratio, CrI—credible interval, RD—risk difference, IVH—intraventricular hemorrhage, CPAP—continuous positive airway pressure, RR—relative risk, CI—confidence interval, CPAP failure—need for mechanical ventilation within 72 h of birth, NNB—number needed to benefit, NICU—neonatal intensive care unit, PTX—pneumothorax, PVL—periventricular leukomalacia.

**Table 3:** Insulin, TSH, and thyroxine content of human preterm milk and donor milk, before and after Holder pasteurization (HoP).

Hormone	Preterm Milk	Donor Milk Row	Donor Milk HoP
Insulin, pg/mL	1396 ± 302 (*n* = 26)	1328 ± 178 * (*n* = 30)	1152 ± 149 * (*n* = 30)
TSH, nU/L	18.4 ± 1.4 (*n* = 90)	20.6 ± 3.3 ** (*n* = 44)	5.4 ± 0.6 ** (*n* = 44)
Thyroxine, nmol/L	671.6 ± 61.2 (*n* = 90)	640.1 ± 32.4 * (*n* = 44)	506.1 ± 11.2 * (*n* = 44)

Data were obtained from the publications of Vass et al., [207,210]. Results are expressed as the mean  ±  SEM * *p* < 0.001, ** *p* < 0.0001.

## Abbreviations

RDS—respiratory distress syndrome, BPD—bronchopulmonary dysplasia, ROP—retinopathy of prematurity, IVH—intraventricular hemorrhage, PVL—periventricular leukomalacia, NEC—necrotizing enterocolitis, LISA-nCPAP—less invasive surfactant administration (LISA) combined with the nasal continuous positive airway pressure (nCPAP) ventilation, GA—gestational age.
